# Nutrient Composition of Popularly Consumed African and Caribbean Foods in The UK

**DOI:** 10.3390/foods8100500

**Published:** 2019-10-15

**Authors:** Tanefa A. Apekey, June Copeman, Nichola H. Kime, Osama A. Tashani, Monia Kittaneh, Donna Walsh, Maria J. Maynard

**Affiliations:** 1 School of Clinical & Applied Sciences, Leeds Beckett University, Leeds LS1 3HE, UK; 2 School of Sport, Leeds Beckett University, Leeds LS6 3QQ, UK

**Keywords:** nutrients, food composition, African, Caribbean, macronutrients, energy, vitamins and minerals

## Abstract

(1) Background: Traditional foods are important in the diets of Black Africans and Caribbeans and, more widely, influence UK food culture. However, little is known about the nutritional status of these ethnic groups and the nutrient composition of their traditional foods. The aim was to identify and analyse African and Caribbean dishes, snacks and beverages popularly consumed in the UK for energy, macronutrients and micronutrients. (2) Methods: Various approaches including focus group discussions and 24-h dietary recalls were used to identify traditional dishes, snacks, and beverages. Defined criteria were used to prioritise and prepare 33 composite samples for nutrient analysis in a UK accredited laboratory. Quality assurance procedures and data verification were undertaken to ensure inclusion in the UK nutrient database. (3) Results: Energy content ranged from 60 kcal in Malta drink to 619 kcal in the *shito* sauce. Sucrose levels did not exceed the UK recommendation for adults and children. Most of the dishes contained negligible levels of *trans* fatty acid. The most abundant minerals were Na, K, Ca, Cu, Mn and Se whereas Mg, P, Fe and Zn were present in small amounts. (4) Conclusion: There was wide variation in the energy, macro- and micronutrients composition of the foods analysed.

## 1. Introduction

In the United Kingdom (UK), as in other high-income countries, nutrition related ill-health is more common in some minority ethnic groups. For example, obesity, type 2 diabetes, hypertension and cardiovascular conditions are more common among ethnic groups of Black African origin compared to the majority population [1,2,3,4].

The development and implementation of effective public health programmes and nutrient recommendations requires reliable data on the nutritional status of the target population. In the UK, as elsewhere, national diet and nutrition surveys are regularly carried out in order to assess the dietary habits and nutritional status of the population. Information from these surveys inform government policies, public health education and interventions to promote nutrition related health and prevent non-communicable diseases [5]. According to the 2011 UK census, about 20% of the population self-identified as non-white British. Those from South Asian groups make up the largest minority ethnic group (7.5%). People of Black ethnicity were the second largest minority group (3.3%), with Black Africans being the fastest growing minority population [6,7]. The UK National Diet and Nutrition Survey (NDNS) which began in 1992 is designed to assess the dietary habits and nutritional status of adults and children [5]. The survey is the only source of high-quality data on dietary intakes and nutritional status in a representative sample of the population [8]. However, minority ethnic groups are not represented in the NDNS and other annual health surveys such as Health Survey for England [5,9,10]. To date, only two national health surveys have been conducted with boosted ethnic minority samples; this was in 1999 and 2004 [9,10]. The data collected involved questionnaire-based interviews, physical measurements, blood sample analysis, health and psychosocial wellbeing, cardiovascular disease (CVD) risk, tobacco use, alcohol consumption, obesity, blood pressure and physical activity and eating habits among the African-Caribbean, South Asian, Chinese and Irish groups throughout England. The data on eating habits was based on a food frequency questionnaire which did not include the traditional foods of these minority ethnic groups. The absence of these traditional foods is mainly due to the lack of reliable and comprehensive data on their nutrient composition. Ethnic foods are becoming increasingly popular and also contribute to the UK food culture They contribute around 19% of foods consumed (at least 4% of which are African and Caribbean foods) [11,12]. A reliable nutrient composition database of these traditional foods is therefore needed for comprehensive assessment of the nutritional status and dietary habits of these population groups. Accurate nutrient data are also essential in monitoring health and nutritional status as well as the development of tailored initiatives to tackle the widening inequalities in health and to improve nutrition related health [13].

The aim of the current study was to identify and analyse African and Caribbean dishes, snacks and beverages popularly consumed in the UK for energy, macronutrients and micronutrients. These new nutrient composition data will have various uses including nutritional surveys and health surveillance in Black ethnic groups and the majority population of the UK. This study is part of the programme of research of the Migrant Health Research group, School of Clinical and Applied Sciences, Leeds Beckett University. One of the aims of the group is to develop reliable and comprehensive nutrient composition data for popular multi-ethnic foods in the UK.

## 2. Materials and Methods

The full details of the methodology have been described elsewhere [14]. The procedures followed in developing these data are in line with the FAO (Food and Agriculture Organisation of the United Nations) and INFOODS (International Network of Food Data Systems) guidelines on production, management and data quality of food composition data [15,16].

Briefly, all volunteers were provided with an information sheet on the study and written consent was obtained; in accordance with the 1975 Declaration of Helsinki. The study was approved by the Faculty of Health and Social Science Research Ethics Committee, Leeds Beckett University (reference number 22,946).

### 2.1. Identification, Selection and Sampling of Popularly Consumed African and Caribbean Foods

Different sources including Mintel reports on ethnic foods and restaurants in UK [17,18], consumption data from food surveys and research papers [19,20,21,22,23,24,25,26,27,28,29,30,31] as well as data from major food retailers including ethnic food retailers, manufacturers, restaurants and takeaways were used to identify popularly consumed North African, West African and Caribbean dishes, snacks and beverages in the UK. Additional new data were collected using 24-h dietary recall, 10 focus group discussions and 5 individual interviews with African (North and West) and Caribbean adult over 18 years, living in Leeds, UK. See Figure S1 for stages involved in the selection of dishes, snacks and beverages for analyses.

A total of 33 (14 West African, 14 Caribbean and 5 North African) dishes, snacks and beverages were prioritised for nutrient analyses. Prioritisation was based on food consumption patterns, common nutrition-related diseases, consumer demand and preference, relevance to health inequalities and data from the focus groups and individual interviews. Traditional desserts are not commonly consumed [19,20,21,22,23,24,25,26,27,28,29,30,31] and therefore were not included. Table 1 shows the description of the 33 prioritised foods (dishes (*n* = 26), snacks (*n* = 3, plantain chips, meat patties and fried dumplings) and dessert (*n* = 1, *kunafa*) and beverages (*n* = 3, ‘Malta’ or other malt drink, rum and Guinness (Irish stout beer) punch).

### 2.2. Sampling, Preparation and Analyses of Prioritised Dishes, Snacks and Beverages

Traditional foods and ingredients were purchased from the four UK supermarkets with the largest market share (Tesco, Sainsbury’s, Asda and Morrison’s) as well as ethnic food shops and stalls, by stratified sampling approach. Stratification was based on type of retail outlet or sale point, sources, location and manufacturer brands. Ingredients were randomly purchased within each stratum in order to account for variations such as manufacturer’s brands, processing conditions and retail outlet.

Female volunteers, 6 West African, 6 North African and 9 Caribbean were recruited to cook the prioritised dishes, beverages and snacks in the Nutrition kitchen at the University. They were recruited from places of worship and recreation, and other local hubs through word of mouth and poster advertisements. They all received an information sheet on the study and written consent was obtained.

The volunteer cooks regularly prepare and consume their assigned traditional dishes, beverages and snacks, as such were familiar with the ingredients, recipes and cooking procedures. Prior to the cooking sessions, the volunteers provided the list of ingredients and recipes, including quantities. Recipe harmonisation was by identification of common recipes, types and quantity of ingredients and methods of food preparation from the sources previously mentioned. These recipes and ingredients matched those provided by the volunteers. Preparation of dishes, snacks and beverages was therefore based on the harmonised recipes.

Composite samples were prepared according to procedures described by Apekey et al. [14]. Equal weights (500 g of edible portions) of similar foods, beverages or snacks were combined by mixing in a food blender to form a composite sample weighing ≈ 4000 g. Composite samples were prepared from 1 to 8 primary samples in order to reflect the variability in the composition due to recipe variations. Rigorous quality assurance procedures and verification of data were undertaken to ensure inclusion in the UK nutrient database, McCance and Widdowson’s The Composition of Foods. A total of 33 samples were sent to a UK accredited laboratory in Leeds for nutrient analyses. See Figure S2 and Figure S3 for composite sample preparation process.

The methods used are accredited through the United Kingdom Accreditation Service (UKAS) to the ISO 17025 (International Organisation for Standardisation) standard and as such are fully validated. In order to meet the repeatability criteria documented in the methods used, the analytical tests were repeated. The analytical methods used are described in Table 2. 

## 3. Results and Discussion

The new data represents the energy, macronutrients (Table 3, Table 4, Table 5, Table 6, Table 7 and Table 8), mineral (Table 9 and Table 10) and vitamin (Table 11, Table 12 and Table 13) composition per 100 g edible portion of Caribbean, North and West African dishes, snacks and beverages popularly consumed in the UK.

### 3.1. Moisture, Energy, Carbohydrate, Protein and Fat Composition

All the foods analysed contained moisture ranging from 4 to 84.8 g/100 g (Table 3, Table 4 and Table 5). The wide variation in the moisture content is attributed to the type of ingredients and cooking method used. *Shito* sauce and plantain chips require deep fat frying which results in a decrease in moisture with a simultaneous increase in oil [32], hence the low moisture content of these two foods.

Calculated energy values (Table 3, Table 4 and Table 5) ranged from 60 kcal in Malta drink to 619 kcal (per 100 g edible portion) in the *shito* sauce. For the *shito* sauce, the ingredient of the highest amount is oil, hence the high energy value recorded. An observational study by Goff et al. [21] reported that in the UK the principal sources of energy in the adult Caribbean diet included *rice and peas* and sugar sweetened beverages, whereas for Ghanaians it was *jollof* rice. These foods are however lower in energy than *shito* sauce in the current study. These new food composition data would allow for better quantification of nutrient intake and recommendation of serving size in these population groups. It would also enable health care professionals to identify which foods to encourage or otherwise, when providing dietary advice. Carbohydrate level ranged from less than 0.1 g (in *jerk* chicken and goat curry) to 62.1 g/100 g edible portion of plantain chips (Table 3, Table 4 and Table 5).

The relationship between dietary carbohydrate intake and risk of hypertension, stroke, type 2 diabetes and obesity, all of which are predominant in people of African and Caribbean ethnicities in the UK [1,2,4] continue to receive a lot of attention. Recently there has been specific focus on carbohydrates and type 2 diabetes. US academics and clinicians are calling for carbohydrate restricted diets as a first approach to prevention and management of type two diabetes [33]. The British Dietetics Association now advise supporting people’s choice of low carbohydrate diets for weight loss and diabetes management [34]. On the other hand, the Scientific Advisory Committee on Nutrition [35] considered evidence from both prospective cohort studies and randomised controlled trials on carbohydrates and health. The committee concluded that total carbohydrate intake appears to be neither detrimental nor beneficial to cardio-metabolic (including cardiovascular disease, insulin resistance, glycaemic response and obesity) health. The main starch containing foods were fried dumplings, salt fish, meat patties, *keneky*, *fufu*, plantain chips, *eba*, *rice and peas*, *jollof* rice and *kunafa* (Table 3, Table 4 and Table 5). The review by SACN [35] reported no association between total starch intake and incidence of coronary events or type 2 diabetes. Corn porridge, *kunafa* and the sugar-sweetened beverages (Malt/Malta, Guinness and rum punch) contained the highest amounts of total sugars. Sucrose levels were mostly less than 1 g but highest in *kunafa* (18.6 g) and Guinness punch (11.7 g). Lactose levels were general less than 0.1 g/100 g of edible portion, therefore negligible (Table 3, Table 4 and Table 5). However, high consumption of sugar-sweetened beverages is associated with type 2 diabetes and weight gain in children and teenagers [36,37].

In addition, a review by SACN [35] indicated that limited intake of free sugars (total of Non Milk Extrinsic Sugars and added sugars) could reduce the risk of heart disease, type 2 diabetes, bowel health and tooth decay hence the recommendation to limit intakes to 19 g or 5 sugar cubes for children aged 4 to 6, 24 g or 6 sugar cubes for children aged 7 to 10 and 30 g or 7 sugar cubes for 11 years and over, based on average population diets. The current food composition data shows that sucrose levels did not exceed the SACN recommendation for both adults and children.

Increased intakes of total dietary fibre, especially cereal fibre and wholegrain are strongly associated with a lower risk of cardio-metabolic disease [35]. Plantain chips contained the highest amount of fibre of 5.5 g/100 g (Table 6, Table 7 and Table 8). Non-starch polysaccharide (NSP) levels ranged from 0.3 to 23.7 g/100 g of edible portion of food (Table 6, Table 7 and Table 8). For those who regularly consumed the dishes, snacks and beverages analysed in the current study, other sources of dietary fibre would need to be included in their diet in order to meet the SACN [35] recommendations (fibre intake of 30 g a day for those aged 16 and over, 25 g for 11 to 15-year-olds, 20 g for 5 to 11-year-olds and 15 g for 2 to 5-year-olds).

The protein content (Table 3, Table 4 and Table 5) of most of the dishes, snacks and beverages apart from rum punch, Malta drink and *eba* was above 1 g with *jerk* chicken containing the highest amount of 27.4 g/100 g. The contributors of protein were from animal, fish and vegetable sources hence the noticeably low levels in the beverages (rum punch and Malta drink) and *eba* which is made from ground cassava. Although the protein composition of the vegetable dishes (e.g., vegetable couscous, 4.3 g of protein/100 g of edible portion) were comparatively lower, current evidence suggests that dietary patterns based on more plant sources of protein, or that include unprocessed animal protein also low in saturated fats, could reduce the risk of cardiovascular diseases [38]. Thus, these new data could provide guidance on cardiovascular health in both the majority and Black ethnic populations in the UK [39].

Total fat includes triglycerides, phospholipids, sterols and related compounds. Only *shito* sauce, plantain chips and *kunafa* contained over 20 g/100 g of fat (Table 6, Table 7 and Table 8). *Shito* sauce, plantain chips, *Egushi* stew and *kunafa* contained over 5 g of saturated fatty acids (SFA)/100 g edible portion of food (Table 6, Table 7 and Table 8). They also contained comparatively high levels of monounsaturated fatty acids, MUFA. Nearly half the samples analysed had less than 1 g of polyunsaturated fatty acids (PUFA) per 100 g of edible portion of food. Furthermore, *trans* fatty acids (TFA) levels were generally less than 1 g per edible portion of food, hence considered negligible. The main fatty acids present in the foods analysed were SFA, MUFA and PUFA. With reference to current nutrition labelling guidance in UK, *shito* sauce, plantain chips, *egushi* stew and *kunafa* would be classified as high fat foods because they contained over 5 g SFA/100 g edible portion of food [40,41]. A key focus of dietary advice and guidelines is the four fatty acids (TFA, SFA, MUFA, n-3 PUFA and n-6 PUFA) because of their reported association with cardiovascular disease risk [42,43,44]. However, the previous notion that dietary SFAs lead to increase in serum cholesterol and thus contribute to the risk of cardiovascular disease risk [45] has been challenged [46]. A review by Hammad et al. [47] found that replacing SFA and TFA with n-6 PUFA, n-3 PUFA, or MUFA might protect cardiovascular health but the optimal amount of PUFA or MUFA that can be used to replace SFA and TFA was not identified.

### 3.2. Mineral Composition

Generally, there were wide variations in the mineral content of the dishes, beverages and snacks analysed. This could be attributed to factors such as variations in ingredients, recipes, cooking or processing methods and brands. The most abundant minerals were Na, K, Ca, Cu, Mn and Se, whereas Mg, P, Fe and Zn were present in small amounts (Table 9 and Table 10). Generally, chloride level was less than 370 mg per 100 g edible portion of all the dishes, beverages and snacks.

Sodium (Na) levels in the dishes, beverages and snacks ranged from 3 to 313 mg/100 g (1 gram of sodium per 100 g = 2.5 grams salt). High salt intake is strongly linked to raised blood pressure which increases the risk of heart disease and stroke; common and major causes of death in Europe and UK [48,49]. Although salt intake in the UK is currently on a steady downward trend, levels are 8 g per day on average, therefore above the recommendation of no more than 6 grams per person per day for adults. A reduction in average salt intake from 8 g to 6 g per day is estimated to prevent over 8000 premature deaths each year and save the UK National Health Service (NHS) over £570 million annually [50]. A review by Van-Horn [51] concluded that recommendations to reduce sodium intakes to 2400 mg/d were beneficial. Thus, these traditional dishes, beverages and snacks would increase the low salt options for consumers, which could lead to reduction in overall daily salt intake.

There is increasing evidence to suggest that lower potassium intake or serum potassium levels are associated with a higher risk for type 2 diabetes [52,53,54]. Although potassium levels (Table 9 and Table 10) were less than the UK recommendation of 3.5 mg/day for adults [55], intervention studies are needed to prove that high intakes or supplementation can improve glucose metabolism.

There is evidence to suggest lower calcium intake below the lower reference nutrient intake (LRNI) in some UK minority groups especially women of Black and Asian ethnicities and living on low income [56]. There was calcium present in all the dishes, beverages and snacks analysed (Table 9 and Table 10). However, to ensure adequate intake, individuals who regularly consume these dishes would need to include other calcium rich foods in their diet. The latest NDNS data shows that mean intakes of vitamin D were below the RNI (reference nutrient intake) in all age/sex groups and therefore at greater risk of developing a deficiency [50]. About 15 minutes daily exposure to sunlight is recommended. Taking a daily supplement of 10 µg vitamin D is also recommended for the UK population, especially ethnic minority groups from African, Afro-Caribbean and South Asian backgrounds with dark skin and /or cover their bodies when outdoors for cultural reasons, who may not get enough exposure to sunlight [50].

In the UK, around 48% of girls 11 to 18 years and women aged 19 to 64 years have iron intakes below the LRNI and with evidence of anaemia [50]. Iron deficiency anaemia has been associated with low offspring birthweight, can increase susceptibility to infection, and also impact on cognitive development of children and adolescents [57,58]. Data from UK dietary surveys including the Low Income Diet and Nutrition Survey (LIDNS) [26,56,59,60,61,62] suggest that iron intakes or status in some South Asian and Black African-Caribbean ethnic minority populations is lower than their White British counterparts. However, according to the SACN [63] report on iron and health, available data suggest that iron intakes of minority ethnic groups aged 16 years and over are not below those of the general UK population. The lack of reliable data on biochemical markers of iron status in UK Black population would account for differences in reported iron intakes and status. The iron content of the dishes, beverages and snacks in the current study were low and ranged from <0.2 to 2.8 mg/100 g edible portion of food (Table 9 and Table 10). Thus, individuals who regularly consume these dishes, beverages and snacks will need to include other sources of iron in their diet to prevent the risk of anaemia.

The zinc content was generally very low (Table 9 and Table 10) and therefore these foods are not adequate sources of this micronutrient. Zinc is required for growth and normal function of the immune system. Although Zn deficiency is associated with poor growth and increased risk of infection, there is no reliable biomarker to identify the status of this micronutrient [64,65].

Selenium was present in each dish, beverage and snack although levels were varied with plantain chips containing the highest amount—94 μg/100 g of edible portion. In the UK, a substantial proportion of adults aged 19 years and over have selenium intake below the LRNI but the health implications of this are unclear [50].

*Jerk* chicken, *callaloo* and saltfish, fried dumplings and *egushi* stew contained higher levels of most of the nutrients but they are high in fat. If adequate portion sizes are consumed, they would provide health benefits especially to these three population groups that have been shown to be vulnerable to inadequate micronutrient intake. It is important to note that the adequacy of micronutrient intakes of individuals who regularly consumed these foods depend on various factors including food preparation method, portion size, frequency of consumption and bioavailability rather than just the mineral content per 100 g of the food. Furthermore, reliable biomarkers are needed for better assessment of micronutrient status.

### 3.3. Vitamins

Vitamin A (Table 11, Table 12 and Table 13**)** was only present in *kunafa* and meat patties (200 µg and 24.3/100 g of food, respectively). However, β-carotene was present in twenty-five of the foods analysed. In addition, results from the NDNS showed that mean daily intake of most vitamins derived from dietary sources were close to or above the RNI [5]. Based on UK recommendation for vitamin A [55], *kunafa* could contribute about a third of the RNI of vitamin A (i.e., representing RNI of about 33% for females, 29% for males (11 years and over); 50% RNI for children age 1 to 10 years). However, this dessert is high in sugar and therefore modified recipe (containing reduced sugar) should be adopted by those who consume it. Vitamin D was not present in any of the foods. People of Black ethnicity are among the groups identified as vulnerable to vitamin D deficiency [50]. It is therefore crucial that, this population group increases their exposure to sunlight and also take supplements to avoid the risk of deficiency since dietary sources are unlikely to meet the current RNI of 10 µg per day [66]. There is growing interest around the bioavailability, metabolism, nonantioxidant activity and the role of the various forms of vitamin E in human diseases. Alpha-and gamma-tocopherols are considered the two major forms of the vitamin depending on the source [67]. European Food Safety Authority, EFSA [68] defined Adequate Intake of alpha-tocopheral as 13 mg/day for men, 11 mg/day for women, 6 mg/day for children aged 1 to <3 years (both sexes), 9 mg/day for children aged 3 to <10 years (both sees), for children aged 10 to <18 years, 13 mg/day for boys and 11 mg/day for girls and for infants aged 7–11 months, this was set at 5 mg/day. *Shito* could contribute adequate amount of alpha-tocopherol to the diet. This is likely to be due to the high PUFA content of this sauce.

The water-soluble vitamin composition of the food also varied. Folate was present in most foods, unlike vitamins C and B12. Folate levels range from 0 to 43.2 µg/100 g which is below the RNI for all age groups. The UK government has launched a consultation in 2019 to consider the practicality and of mandatory folic acid fortification, along with the controls on voluntary fortification [69]. The absence or low levels of vitamin C may be attributable to heat losses during cooking. Groundnut soup and goat curry contained the highest amount of biotin (16.1 µg/100 g) and vitamin B12 (2.13 µg/100 g), respectively. Similarly, the highest concentration of calcium pantothenate (1.25 mg/100 g) and vitamin B5 (1.15 mg/100 g) were found in *jerk* chicken and vitamin B1 (0.297 mg/100 g) in Malta drink. Guinness punch had the most amount of vitamins B2 (0.295 mg/100 g) and B6 (0.231 mg/100 g).

### 3.4. Comparison with Similar Foods in the UK Nutrient Database (McCance and Widdowson’s the Composition of Foods)

The only similar food identified in McCance and Widdowson’s The composition of foods [70] was ripe plantain, fried in vegetable oil. This was different in composition to the plantain chips in the current data. For instance, the moisture content was higher (34.7 g vs. 4 g), fat lower (9.2 g vs. 24.2 g) and lower energy (267 kcal vs. 484 g) in the ripe plantain, fried in vegetable oil compared to the plantain chips in the current study. The differences would be due to the cooking method, variety of plantain and degree of ripening. In addition, fried plantain is usually consumed as part of a dish whereas plantain chips are snacks. Furthermore, plantain chips in the current study are prepacked samples that are thinly-sliced and deep fat fried to reduce moisture content and enhance shelf life. Foods such as *rice and peas* and *jerk* chicken are very popularly consumed among both the majority and ethnic minority populations in the UK [19,20,21,22,23,24,25,26,27,28,29,30,31], but nutrient data for these foods are not available in the McCance and Widdowson food composition tables [70].

### 3.5. Strengths and Limitations

The study team comprised of trained researchers (including a food scientist and registered nutritionists) with experience in food composition and analyses, and who are of African or Caribbean ethnicity. As previously described by Apekey et al. [14], the various sources, approaches and interview probing questions used enabled the identification of popularly consumed foods. The use of focus group interviews and 24-h dietary recalls also enabled the identification of foods regularly consumed, determination of the frequency of consumption over a period and also improved precision. The interviews and focus group discussions lasted for an hour and therefore allowed for the researchers to capture detailed information on traditional foods, recipes, cooking methods and frequency of consumption. The use of volunteers of the relevant ethnicities in the food preparation in a university Nutrition kitchen allowed for variations in recipes and cooking methods to be taken into account, thereby enhancing the authenticity of the dishes, beverages and snacks. Furthermore, the food samples were analysed in a UK accredited laboratory with trained staff and rigorous quality assurance procedures were followed to ensure the data obtained is reliable and valid.

Limitations of the study include possible introduction of selection bias by the use of convenient sampling to recruit volunteer. However, this approach of sampling through community partnerships or organisations has been shown to improve recruitment of minority ethnic groups into health-related research [71]. The use of 24-h recall may introduce recall bias since it relies on the memory of the volunteers [72]. Although analysing individual foods instead of composite ones may improve the representativeness of the samples analysed, this approach is more complex, time consuming [73] and beyond the scope of the present study.

### 3.6. Implications for Future Research and Practice

These new nutrient data will contribute to ongoing interactive educational workshops with local communities, and nutrition education and resources in diabetes clinics. The data will allow for better quantification of nutrient intake and recommendations for appropriate serving sizes in these population groups. Furthermore, they will also enable health care professionals to identify which foods to encourage or otherwise, when providing dietary advice. The data will be made available to international and relevant European agencies, for inclusion in their Nutrient Databanks such as the UK’s McCance and Widdowson’s The Composition of Foods. It will also be made available to health authorities, policy makers and other bodies that have direct influence on promoting health and wellbeing. The data will have various potential uses including (1) contribution to the evidence base of food habits and diet quality, of direct value to nutritional surveys and health surveillance in African and Caribbean populations in the UK and elsewhere in Europe, (2) provide information for components of health promotion programmes contributing to addressing health inequalities and improving quality of life, (3) provide accurate energy and nutrient composition of key dishes for more reliable nutrition labelling and (4) further contributing to health promotion, and food composition data, and the nutrition, dietetics and public health curriculum in the UK and elsewhere.

## Figures and Tables

**Table 1:** Description and proportion of ingredients for the prioritised dishes, snacks and beverages.

Ethnic Group	Food	Food Description
1. Caribbean	Rice and peas	Rice (19%) boiled in water (26%) and combined with black eyed, split or pigeon peas or kidney beans (26%); onions (0.5%), vegetable oil (2%), salt (0.5%) and coconut cream (26%) may be added.
2. Caribbean	Ackee and saltfish	Tinned ackee 38% (a tropical fruit, yellow in colour), saltfish (40%), onion (0.5%), garlic (0.5%), red/yellow pepper (0.5%), chopped tomato (19%), curry power/jerk seasoning (0.5%) and spring onion (0.5%).
3. Caribbean	West Indian soup	Made with meat (14%), dumplings (14%), large pieces of vegetables such as yam (7%), sweet potato (7%), pumpkin (7%), carrots (7%), noodle (7%) and chocho (a green tropical fruit, 11%) in thin stock, curry (5%) and water (21%).
4. Caribbean	Goat curry	Goat meat (65%) usually seasoned overnight with curry powder (2%), onions (0.5%), ginger (1%), cloves (0.5%) and scotch bonnet chillies (1 to 2) and then fried in oil (10%), water (20%) is added and left to cook until tender. Coconut cream (18%) and tomato puree (2%) may be added, with less water.
5. Caribbean	Jerk chicken	Chicken wings (80%), onions (2.5%), pepper (2.5%) and jerk sauce (marinade made with hot spices, 15%).
6. Caribbean	Caribbean fish curry	Headless red/white fish/ haddock (58%), coconut milk (20%), garlic (0.5), thyme (0.5%), carrots (2%), curry powder (2%), tomato (11%), spring onion (2%), onions (2%) and knob of butter (2%).
7. Caribbean	Caribbean vegetable curry	Red/white onion (2%), broccoli (8%), courgetti (8%), butternut squash (8%), cauliflower (8%), carrots (8%), green beans (8%), aubergines (8%), tomato (8%), thyme (0.5%), coconut milk (30%), curry powder (2.5%) and knob of butter (1%).
8. Caribbean	Callaloo and saltfish	Tinned callaloo (a green tropical fruit, 45%), saltfish (28%), water (16%), onion (2%), garlic (2%), carrots (3%), red/yellow pepper (2%) and spring onion (2%).
9. Caribbean	Cornmeal porridge	Hot milk (71%) and cornmeal flour (24%) (about 1.4% sweetened condensed milk may be added) flavoured with fresh nutmeg (0.3%), salt (0.3%), sugar (1%) and vanilla (2%). Cinnamon sticks or powder may also be added for flavour.
10. Caribbean	Guinness punch	Guinness (Irish stout beer, 36%), sweetened condensed milk (21%), vanilla extract (0.4%), cinnamon (0.3%), whole milk (42%) and nutmeg (0.3%).
11. Caribbean	Rum punch	White rum (18%), dark rum (10%), syrup (1.5), lemon/lime (4.5%), water (33%) and pineapple juice (33%).
12. Caribbean	Fried dumplings Also called ‘Johnny cake’	Deep-fried (oil 20%) dough made with white flour (40%), nutmeg or vanilla extract (0.5), sugar (3%), butter (3%), salt (0.5%), water (32%) and baking powder (1%). Cornmeal may be added.
13. Caribbean	Saltfish fritters	Deep fried batter with saltfish/salted cod (30%), which is purchased dried and soaked overnight to remove salt or boiled to rehydrate, self-raising flour (23%), Scotch bonnet chilli (1%) and cooking oil (46%).
14. Caribbean	Meat patties	Semi-circular or oval shaped pastry [made with all-purpose flour (35%), water (9%), butter/vegetable shortening (5%), salt (0.2%)] filled with seasoned minced beef [made with ground beef (45%), chilli pepper (2%), onion (2%), thyme (0.8%) and garlic (1%)]. Vegetables may be added.
15. West African	Kenkey*^◊^	Fermented corn dough made into a ball, wrapped in corn husk and cooked over heat.
16. West African	Shito sauce ^◊^	Chilli/spicy Ghanaian sauce made with vegetable oil, onion, ginger, tomatoes, dried chilli, smoked fish, smoked shrimps, stock cube and spices.
17. West African	Cassava and plantain fufu*^◊^	Cassava, plantain and potato flour which may contain E102, E110, E450, E471 and/or E321
18. West African	Malt/Malta drink^◊^	Water, barley malt, glucose syrup/sugar, carbon dioxide, colour (E150c), acid (citric acid), liquorice, nicotinamide, pantothenol, thiamin hydrochloride, sodium, riboflavin, phosphate and pyridoxin chloride
19. West African	Plantain chips (chilli and plain) ^◊^	Ripe plantain, vegetable oil, sea salt, powdered chilli, spices, citric acid as a flavour enhancer.
20. West African	Eba (also known as Gari)	Ground cassava (80%) and water (20%).
21. West African	Rice and peas/beans	Black eye/brown bean (26%), long grain/basmati rice (20%), salt (1%) and water (53%).
22. West African	Jollof rice	Long grain/basmati rice (40%), tomatoes (27%), vegetable oil (5%), salt (0.5%), beef and/or chicken stock (10%), chicken/beef (10%), curry (1%), thyme (1%), onions (1%), ginger (0.5%), carrot (2%), Maggi chicken cube (0.5%), garlic (0.5%) and hot red pepper, or scotch bonnet/chilli pepper (1%). Other vegetables, chicken and/or beef may be added.
23. West African	Egushi stew	Egushi (ground melon seeds, 20%), beef stock (5%), stock fish (8%), dried fish (8%), beef (8%), salt (0.2), onions (1%), ugu leaf (fluted pumpkin leaves, 9.6%), Maggi chicken cube (0.2), palm oil (5%), garlic and tomato (35%).
24. West African	Groundnut soup	Peanut butter (15%), tomato (30%), tomato puree (0.8%), scotch bonnet (0.1%), Maggi chicken cube (0.3%), beef (15%), goat (15%), fish (15%), ginger (0.2%), vegetables (7.4%, optional - okro/okra, garden eggs), onions (0.8%) and salt (0.4%).
25. West African	Meat stew	Tomato (43%), tomato puree (9%), beef (37%), vegetable oil (6%), Maggi cube (0.5%), salt (0.5%), scotch bonnet/chilli pepper (0.2%), onions (1%), curry (2%), thyme (0.4%) and ginger (0.4%).
26. West African	One pot pepper/light soup	Soup prepared with vegetables (tomato (20%), tomato puree (4%), scotch bonnet (0.2%), ginger (0.3%), garlic (0.3%)], meat (15%), goat (15%), chicken (15%) and fish (15%), salt (0.2%), cow foot (5%), Maggi stock cube (0.2%), thyme (0.2%) [optional—okra/okra (4.8%) and/or garden egg (4.8%) also known as eggplant)
27. West African	Okro soup/stew	Okro (32%), tomato (21%), scotch bonnet/chilli pepper (0.2%), ginger (0.3%), garlic (0.2%), palm/vegetable oil (6%), beef (17.5%), fish (17.5%), spinach (5%), Maggi stock cube (0.3%).
28. West African	Ewedu soup	Ewedu leaves (33%, jute leaves), cray fish (5.8%), Maggi stock cube (0.5%), salt (0.5%), water (60%) and powdered potash (0.2%).
29. North African	Couscous with chicken	Couscous (25%), chicken (35%), water (20%), onion (0.5%), oil (3%), tomato (5%), mixed spices (5%), carrots (3%), chilli pepper (0.3%), coriander (0.2%) and chickpeas (3%).
30. North African	Couscous with vegetables	Onion (1%), chickpea (3%), tomato (2%), tomato paste (0.8%), mixed spices (1.3%), water (26%), chilli pepper (0.1%), salt (0.2%), parsley/parsley (0.3%), oil (3%), potato (5.3%), carrots (7.8%), cabbage (7.8%), turnip (7.8%), butternut (7.8%),, pumpkin (7.8%),, couscous and (18%).
31. North African	Couscous with lamb	Onion (0.5%), lamb (36%), chilli pepper (0.3%), butternut (5%), parsley (0.2%), ghee (4%), tomato paste (3%), potato (5%), carrots (5%), squash (5%), couscous (31%), water and mixed spices (5%).
32. North African	Traditional Libyan soup (*Sharba* Libiya)	Lamb (24%), onion (1%), tomato (2%), vegetable oil/ghee (14%), herbs (6%), tomato paste (1%), soup pasta (22%), salt, chickpea (23%), cinnamon sticks (0.5%) cardamom pods (0.5%), bay leaf (0.5%) and mixed spices (5.6%).
33. North African	Kunafa (sugar-soaked pastry)	Kunafa pastry(22%), walnuts (20%),, butter (9%), sugar (15%), raisins (5%), water (10%), lemon (0.2%), vanilla (0.3%), cinnamon (0.5%) and extra thick cream (18%).

Food is used in the table to represent dishes, beverages or snacks. *Modified foods i.e., ingredients and or recipes and cooking methods modified to align with UK tastes. **^◊^** Dishes, snacks and beverages did not require cooking.

**Table 2:** Analytical methods used for the nutrient analysis.

Nutrient	Reference Method
Ash	BS4401-1 1998 ISO 936:1998
Moisture	BS4401-3:1997 ISO 1442:1997
Nitrogen (Total nitrogen)	Elementar Rapid N Cube Condensed Manual
Fatty acids by FAME Profile (MUFA, PUFA, SFA, Trans)	Kirk, R S, Sawyer, R, Pearson’s Composition and Analysis of Foods, 9th edn, Longman, 1991, p24
Sugars	The sample is dissolved in water, with heating, clearing and dilution if necessary, and analysed by high performance liquid chromatography using refractive index detection
Chloride	The test sample is extracted in hot water, filtered and analysed by ion chromatography
K, Ca, Mg, P, Fe, Cu, Zn, Cl, Mn, Se	The samples are digested using a digiprep digestion block and analysed by ICP-MS
Sodium	The sample is ashed, the ash dissolved in water and the sodium content determined by Flame Photometry
Dietary fibre (AOAC)	AOAC method 985 29
Dietary fibre (NSP)	The sample undergoes enzymatic hydrolysis of starch, precipitation of NSP in ethanol, acid hydrolysis of the NSP and measurement of the released constituent sugars (Englyst)
Energy	Calculated from the protein, fat, carbohydrate (including sugars), and AOAC fibre using the values in Annex XIV of Regulation (EU) No 1169/2011. No allowance was made for the presence of any polyols, salatrims, alcohol, organic acid or erythritol
Protein	Total nitrogen multiplied by 6.25
Total fat	BS:4401:Part 4 1970
Carbohydrate	Available carbohydrate is calculated by difference (100 minus the sum of protein, total fat, ash, moisture, alcohol and AOAC fibre) in 100 g of food
Vitamin A	A7272: Vitamin A (Retinol). EN 12823-1 2014, LC-DAD
ß-carotene	A7270: Beta-carotene, juice and vegetables. Pro-vitamin A; EN 12823-2:2000, LC-DAD
Vitamin B1 thiamine base	A7273: Vitamin B1 -Thiamine base. EN 14122:2003, mod., LC-FLD, Food and feed
Vitamin B12	DJCDE: Vitamin B12 HPLC (Immuno) Food and Feed. J. AOAC 2008, vol 91 No 4, LC-UV/DAD
Vitamin B2 (riboflavin)	A7274: Vitamin B2—riboflavin; EN 14152:2003, mod. LC-FLD
Vitamin B5 (Panthotenic acid)	DJ5BG: Vitamin B5 LC/MS/MS; AOAC 2012.16, LC/MS/MS with isotope dilution.
Biotin (vitamin H)	A7284: Vitamin B8—biotin, microbiological. Analogous to FDA method, LST AB 266.1,1995, Nephelometry
Vitamin B6 (pyridoxine)	A7251: Vitamin B6. EN 14164, LC-FLD
Calcium Pantothenate	See Vitamin B5 (Panthotenic acid)
Vitamin C	A7291: Vitamin C. Food Chemistry, 94 626-631, LC-DAD
Vitamin D2	A7294: Vitamin D2 (µg/100g). EN 12821:2009, LC-DAD
Folic acid total (vitamin B9/M)	A7286: Vitamin B9—Total folate, microbiological; NMKL 111:1985, Nephelometry
α, β, γ and δ Tocopherol	See sum of tocopherols
Sum tocopherols	A7297: Vitamin E (tocopherol profile). EN 12822:2014, LC-FLD
Vitamin PP/B3	DJB05: Vitamin B3 (Total Niacin) EN-HPLC; EN 15652:2009, LC-FLD.

K—Potassium; Ca—Calcium; Mg—Magnesium, P—Phosphorus; Fe—Iron; Cu—Copper; Zn—Zinc; Cl—Chlorine; Mn—Manganese; Se—Selenium; MUFA—Monounsaturated fatty acids; PUFA—Polyunsaturated fatty acids; SFA—Saturated fatty acid; NSP—Non-starch polysaccharide; α—alpha; β—beta; γ—gamma and δ-delta.

**Table 3:** Energy, protein, carbohydrate and moisture composition of Caribbean dishes, snacks and beverages in the UK (per 100 g edible portion).

Dish/Snack/Beverage	Moisture (g)	Total Nitrogen (g)	Protein (g)	Carbo-Hydrate (g)	Energy		Starch (g)	Total Sugars (g)	Individual Sugars (g)				
					Kcal	kJ			Gluc	Fruct	Sucr	Malt	Lact
**1. Rice and peas**	**65.7**	**0.79**	**4.9**	**24.8**	**131.0**	556.0	24.1	0.7	0.1	0.1	0.6	<0.1	<0.1
2. Ackee and saltfish	66.6	1.58	9.9	3.5	203.0	842.0	<0.1	3.5	0.9	1.1	1.5	<0.1	<0.1
3. West Indian Soup	79.1	0.86	5.4	9.9	87.0	364.0	7.6	2.2	0.5	0.4	1.1	0.3	<0.1
4. Goat Curry	66.6	2.99	18.7	<0.1	172.0	717.0	<0.1	1.9	0.5	0.5	0.6	0.2	<0.1
5. Jerk chicken	60.3	4.39	27.4	<0.1	195.0	817.0	<0.1	2.2	0.2	0.3	1.1	0.4	0.3
6. Caribbean fish curry	76.9	1.65	10.3	3.6	100.0	420.0	<0.1	3.6	1.0	1.1	1.2	0.3	<0.1
7. Caribbean vegetable curry	84.8	0.32	2.0	7.4	65.0	271.0	2.4	5.0	1.4	1.3	2.2	<0.1	<0.1
8. Callaloo and Saltfish	72.5	1.32	8.3	4.3	141.0	585.0	<0.1	4.3	1.1	1.1	2.1	<0.1	<0.1
9. Cornmeal porridge	69.3	0.62	3.9	24.0	125.0	528.0	13.3	10.8	<0.1	<0.1	6.7	<0.1	4.1
10. Guinness Punch	81.4	0.50	3.1	15.8	106.0	446.0	<0.1	15.8	0.2	<0.1	11.7	<0.1	3.9
11. Rum punch	73.6	0.01	0.1	26.1	106.0	450.0	11.2	14.9	5.2	5.0	4.7	<0.1	<0.1
12. Fried Dumplings	31.1	0.93	5.8	47.3	318.0	1336.0	46.0	1.3	0.0	0.0	0.2	0.9	0.2
13. Saltfish fritters	49.0	1.51	9.4	24.3	261.0	1093.0	21.9	2.4	0.5	0.4	0.4	0.8	0.4
14. Meat patties	33.9	1.90	11.9	37.4	311.0	1304.0	34.1	3.3	0.4	0.4	0.8	1.7	<0.1

**Table 4:** Energy, protein, carbohydrate and moisture composition of West African dishes, snacks and beverages in the UK (per 100 g edible portion).

Dish/Snack/Beverage	Moisture (g)	Total Nitrogen (g)	Protein (g)	Carbo-Hydrate (g)	Energy		Starch (g)	Total Sugars (g)	Individual Sugars (g)				
					Kcal	kJ			Gluc	Fruct	Sucr	Malt	Lact
**15. Kenkey**	**69.3**	**0.42**	**2.6**	**22.5**	**118.0**	498.0	22.2	0.3	0.3	<0.1	<0.1	<0.1	<0.1
16. Shito sauce	17.6	0.79	4.9	14.2	619.0	2555.0	11.2	3.0	0.7	1.3	1.0	<0.1	<0.1
17. Cassava and Plantain fufu	77.4	0.22	1.4	18.9	86.0	366.0	18.8	0.2	<0.1	<0.1	0.2	<0.1	<0.1
18. Malt/Malta drink	84.8	0.06	0.4	14.7	60.0	257.0	3.2	11.6	0.9	0.4	6.5	3.7	<0.1
19. Plantain chips (ripe and chill)	4.0	0.27	1.7	62.1	484.0	2024.0	51.7	10.4	0.7	1.0	8.7	<0.1	<0.1
20. Eba (Gari)	71.0	0.03	0.2	26.3	112.0	474.0	26.0	0.2	0.2	<0.1	<0.1	<0.1	<0.1
21. Rice and Peas/Beans	68.6	0.71	4.4	20.5	118.0	498.0	19.9	0.6	0.1	0.1	0.4	<0.1	<0.1
22. Jollof Rice	66.0	2.50	1.6	23.9	155.0	650.0	22.2	1.7	0.7	0.7	0.3	<0.1	<0.1
23. Egushi Stew	63.1	2.54	15.9	2.4	218.0	904.0	<0.1	2.4	0.7	0.6	0.7	0.4	<0.1
24. Groundnut Soup	73.1	1.69	10.6	3.3	144.0	597.0	1.0	2.3	0.5	0.6	1.1	0.1	<0.1
25. Meat stew	71.3	1.41	8.8	5.0	165.0	684.0	<0.1	5.0	2.1	2.1	0.6	0.2	<0.1
26. One pot pepper soup	78.1	2.24	14.0	1.9	93.0	389.0	<0.1	1.9	0.5	0.6	0.6	0.2	<0.1
27. Okro soup/stew	78.5	2.28	7.3	1.8	120.0	498.0	<0.1	1.8	0.6	0.6	0.5	0.1	<0.1
28. Ewedu soup	75.8	1.91	11.9	1.6	112.0	468.0	<0.1	1.6	0.5	0.5	0.3	0.2	<0.1

**Table 5:** Energy, protein, carbohydrate and moisture composition of North African dishes, snacks and beverages in the UK (per 100 g edible portion).

Dish/Snack/Beverage	Moisture (g)	Total Nitrogen (g)	Protein (g)	Carbo-Hydrate (g)	Energy		Starch (g)	Total Sugars (g)	Individual Sugars (g)				
					Kcal	kJ			Gluc	Fruct	Sucr	Malt	Lact
29. Chicken Couscous	74.8	1.13	7.1	11.0	**115.0**	480.0	8.3	2.6	0.8	0.7	0.8	0.4	<0.1
30. Vegetable Couscous	68.0	0.68	4.3	18.6	137.0	577.0	12.8	5.9	2.7	2.8	<0.1	0.4	<0.1
31. Lamb Couscous	67.1	1.08	6.8	18.8	141.0	594.0	16.5	2.3	0.5	0.5	1.1	0.3	<0.1
32. Traditional Libyan Soup	78.4	1.29	7.9	1.7	135.0	560.0	<0.1	1.7	0.6	0.7	0.2	0.2	<0.1
33. Kunafa	27.9	0.46	2.9	42.3	413.0	1721.0	22.5	19.8	<0.1	<0.1	18.6	0.5	0.8

**Table 6:** Fibre and lipid composition of Caribbean dishes, snacks and beverages in the UK (per 100 g edible portion).

Dish/Snack/Beverage	NSP (g)	AOAC Fibre (g)	Fat (g)	SFA (g)	MUFA (g)	PUFA (g)	Trans Fatty Acids (g)
1. Rice and peas	1.9	2.9	0.8	0.3	0.2	0.1	<0.1
2. Ackee and saltfish	1.8	2.3	16.1	4.0	9.0	2.4	<0.1
3. West Indian Soup	1.3	2.0	2.4	1.0	0.9	0.4	<0.1
4. Goat Curry	1.5	2.0	10.4	3.1	5.2	1.4	0.2
5. Jerk chicken	__	__	9.5	2.3	4.5	2.3	<0.1
6. Caribbean fish curry	1.9	3.0	4.3	1.8	1.6	0.7	<0.1
7. Caribbean vegetable curry	2.1	1.1	2.8	0.7	1.0	0.9	<0.1
8. Callaloo and Saltfish	2.2	3.0	9.4	1.7	5.1	2.2	<0.1
9. Cornmeal porridge	0.5	0.9	1.3	0.8	0.3	<0.1	<0.1
10. Guinness Punch	__	__	3.4	2.2	0.8	0.1	<0.1
11. Rum punch	0.3	0.1	0.1	<0.1	<0.1	<0.1	<0.1
12. Fried Dumplings	2.7	2.6	11.2	1.2	6.4	3.0	<0.1
13. Saltfish fritters	2.5	2.2	13.5	1.3	8.0	3.6	<0.1
14. Meat patties	2.3	3.0	12.0	4.0	5.4	2.0	<0.1

__: Food not analysed for the selected nutrient because it was not considered to be an important source of the nutrient.

**Table 7:** Fibre and lipid composition of West African dishes, snacks and beverages in the UK (per 100 g edible portion).

Dish/Snack/Beverage	NSP (g)	AOAC Fibre (g)	Fat (g)	SFA (g)	MUFA (g)	PUFA (g)	Trans Fatty Acids (g)
15. Kenkey	2.2	3.3	1.2	0.2	0.4	0.5	<0.1
16. Shito sauce	__	__	60.3	19.8	27.1	10.5	0.2
17. Cassava and Plantain fufu	0.8	1.4	0.2	0.1	0.1	<0.1	<0.1
18. Malt/Malta drink	__	__	<0.1	<0.1	<0.1	<0.1	<0.1
19. Plantain chips (ripe and chill)	2.1	5.5	24.2	10.8	10.9	1.3	0.1
20. Eba (Gari)	1.1	2.0	0.2	<0.1	0.1	<0.1	<0.1
21. Rice and Peas/Beans	1.3	4.5	1.0	0.2	0.4	0.4	<0.1
22. Jollof Rice	0.8	1.7	5.5	0.8	2.8	1.7	<0.1
23. Egushi Stew	1.5	2.6	15.5	7.2	5.7	1.9	<0.1
24. Groundnut Soup	1.6	2.1	9.3	2.1	6.1	0.7	<0.1
25. Meat stew	1.7	2.4	11.6	1.5	7.1	2.5	<0.1
26. One pot pepper soup	0.7	1.2	3.0	1.3	1.3	0.2	<0.1
27. Okro soup/stew	1.5	2.7	8.7	3.9	3.5	0.9	<0.1
28. Ewedu soup	2.3	2.3	5.9	2.4	2.4	0.8	<0.1

__: Food not analysed for the selected nutrient because it was not considered to be an important source of the nutrient.

**Table 8:** Fibre and lipid composition of North African dishes, snacks and beverages in the UK (per 100 g edible portion).

Dish/Snack/Beverage	NSP (g)	AOAC Fibre (g)	Fat (g)	SFA (g)	MUFA (g)	PUFA (g)	Trans Fatty Acids (g)
29. Chicken Couscous	1.5	2.0	4.3	1.2	1.5	1.4	<0.1
30. Vegetable Couscous	2.3	3.6	4.3	1.5	1.4	1.2	<0.1
31. Lamb Couscous	2.1	2.7	3.7	1.1	1.2	1.1	<0.1
32. Traditional Libyan Soup	1.0	1.2	10.4	4.5	3.5	1.2	0.8
33. Kunafa	0.9	1.0	25.5	13.2	7.0	3.6	0.6

**Table 9:** Inorganic constituents of Caribbean dishes, snacks and beverages in the UK (per 100 g edible portion).

Dish/Snack/Beverage	Na (mg)	K (mg)	Ca (mg)	Mg (mg)	P (mg)	Fe (mg)	Cu (mg)	Zn (mg)	Cl (mg)	Mn (mg)	Se (μg)
1. Rice and peas	15	15	61	0.4	0.2	0.3	345	0.2	<370	15	15
2. Ackee and saltfish	51	28	51	0.8	0.2	0.3	1280	0.2	<370	51	28
3. West Indian soup	32	15	65	0.5	<0.1	0.4	470	0.1	<370	32	15
4. Goat curry	42	28	160	3.0	0.2	2.8	1000	0.2	<370	42	28
5. Jerk chicken	120	32	261	1.4	0.1	2.2	1430	0.1	<370	120	32
6. Caribbean fish curry	35	24	93	1.0	<0.1	<0.2	1250	0.2	<370	35	24
7. Caribbean vegetable curry	33	20	267	0.9	0.1	<0.2	780	0.3	<370	33	20
8. Callaloo and saltfish	115	32	40	1.2	<0.1	0.2	1250	0.3	<370	115	32
9. Cornmeal porridge	97	14	93	<0.4	<0.1	0.2	100	<0.1	<370	97	14
10. Guinness punch	124	13	96	0.6	<0.1	0.2	110	0.1	<370	124	13
11. Rum punch	6	4	3	0.4	<0.1	<0.2	10	0.3	<370	6	4
12. Fried dumplings	256	14	329	1.3	<0.1	0.3	380	0.4	<370	256	14
13. Saltfish fritters	92	15	85	0.9	0.1	0.5	620	0.3	<370	92	15
14. Meat patties	78	21	113	1.9	0.1	2.0	740	0.4	<370	78	21

**Table 10:** Inorganic constituents of African dishes, snacks and beverages in UK (per 100 g edible portion).

Dish/Snack/Beverage	Na (mg)	K (mg)	Ca (mg)	Mg (mg)	P (mg)	Fe (mg)	Cu (mg)	Zn (mg)	Cl (mg)	Mn (mg)	Se (μg)
**West Africa**											
15. Kenkey	11	29	72	2.2	<0.1	0.9	580	0.2	<370	11	29
16. Shito sauce	63	35	81	3.6	0.2	0.7	1210	0.9	<370	63	35
17. Cassava and plantain fufu	11	12	32	<0.4	<0.1	<0.2	30	0.1	<370	11	12
18. Malt/Malta drink	3	6	16	<0.4	<0.1	<0.2	20	<0.1	<370	3	6
19. Plantain chips (ripe and chill)	13	94	84	1.2	0.3	0.5	560	0.5	<370	13	94
20. Eba (Gari)	20	1	13	0.6	<0.1	<0.2	70	0.1	<370	20	1
21. Rice and peas/beans	16	24	71	0.8	0.1	0.6	315	0.3	<370	16	24
22. Jollof Rice	15	11	48	0.8	0.1	0.4	620	0.2	<370	15	11
23. Egushi Stew	313	64	306	3.6	0.3	2.3	920	0.7	<370	313	64
24. Groundnut soup	26	41	118	1.6	0.2	1.8	540	0.3	<370	26	41
25. Meat stew	24	20	79	1.7	0.1	2.1	790	0.2	<370	24	20
26. One pot pepper soup	21	15	87	2.2	0.1	2.2	880	0.1	<370	21	15
27. Okro soup/stew	51	29	82	1.8	0.1	1.2	820	0.1	<370	51	29
28. Ewedu soup	76	27	102	3.6	0.2	1.9	900	0.5	<370	76	27
**North Africa**											
29. Chicken couscous	20	20	82	0.5	0.1	0.5	260	0.2	<370	20	20
30. Vegetable couscous	33	25	83	0.8	0.2	0.5	230	0.5	<370	33	25
31. Lamb couscous	30	32	114	1.1	0.2	1.2	340	0.6	<370	30	32
32. Traditional Libyan soup	24	15	68	0.9	0.1	1.1	340	0.2	<370	24	15
33. Kunafa	53	9	49	0.4	<0.1	0.2	80	0.2	<370	53	9

**Table 11:** Vitamin composition of Caribbean dishes, snacks and beverages in the UK (per 100 g edible portion).

**Sample Dish/Snack/Beverage**	**α-Tocopherol (mg)**	**β-Tocopherol (mg)**	**Biotin (µg)**	**Calcium Pantothenate (mg)**	**δ-Tocopherol (mg)**	**Folic Acid Total (µg)**	**γ-Tocopherol (mg)**	**β-Carotene (µg)**	**Sum Tocopherols (mg)**	**Vit A (µg)**
1. Rice and peas	0	0	1.61	0.154	0	11.4	0	0	0	NA
2. Ackee and saltfish	1.85	0	4.41	0.0816	0	23	1.23	92.2	3.08	0
3. West Indian soup	0.513	0	3.52	0.309	0	16.1	0	310	0.513	0
4. Goat curry	0.734	0	4.94	0.325	0	11.8	0.627	74.2	1.36	0
5. Jerk chicken	1.82	0	12.5	1.25	0	22.2	0	78.4	1.82	0
6. Caribbean fish curry	1.58	0	3.54	0.15	0	17.9	0	2740	1.58	0
7. Caribbean vegetable curry	0.923	0	4.43	0.278	0	20.9	0	629	0.923	NA
8. Callaloo and saltfish	2.4	0	4.75	0.0764	0	16.7	2.24	612	4.64	0
9. Cornmeal porridge	0	0	3.03	0.389	0	7.11	0	15.6	0	NA
10. Guinness punch	0	0	1.91	0.438	0	38.3	0	47	0	NA
11. Rum punch	0	0	0	0.0151	0	8.01	0	111	0	NA
12. Fried dumplings	1.03	0	1.59	0.197	0	9.59	1.88	0	2.91	NA
13. Saltfish fritters	1.85	0	2.41	0.223	0	24.6	2.25	0	4.1	0
14. Meat patties	0.767	0	2.64	0.308	0	22.9	0.879	11.8	1.65	24.3
**Sample Dish/Snack/Beverage**	**Vit B1 (mg)**	**Vit B12 (µg)**	**Vit B2 (Riboflavin) (mg)**	**Vit B5 (Panthotenic Acid) (mg)**	**Vit B6 (Pyridoxine) (mg)**	**Vit C (mg)**	**Vit D2 (µg)**	**Vit PP/B3, (mg)**		
1. Rice and peas	0.064	0	0	0.142	0	0	NA	0.24		
2. Ackee and saltfish	0.018	1.01	0.0436	0.0751	0.0692	2.62	0	0.446		
3. West Indian soup	0.139	0	0.0261	0.284	0.0558	2.26	0	1.08		
4. Goat curry	0.032	2.13	0.0678	0.299	0.0683	0	0	2.83		
5. Jerk chicken	0.094	0.634	0.112	1.15	0.082	NA	0	5.69		
6. Caribbean fish curry	0.03	0.833	0.0284	0.138	0.073	5.76	0	1.04		
7. Caribbean vegetable curry	0.028	0	0.0512	0.256	0.0662	0	NA	0.397		
8. Callaloo and saltfish	0.015	0.884	0.0453	0.0703	0.0844	0	0	0.362		
9. Cornmeal porridge	0.027	0.376	0.137	0.358	0	0	NA	0.134		
10. Guinness punch	0.085	0.629	0.295	0.403	0.121	NA	NA	1.02		
11. Rum punch	0	0	0	0.0139	0	0.829	NA	0.335		
12. Fried dumplings	0.181	0	0	0.181	0	NA	NA	0.854		
13. Saltfish fritters	0.119	0.817	0	0.205	0.0513	NA	0	0.563		
14. Meat patties	0.201	0.729	0.0482	0.283	0.0688	0	0	1.66		

NA—Not Analysed.

**Table 12:** Vitamins composition of West African dishes, snacks and beverages in the UK (per 100 g edible portion).

**Sample Dish/Snack/Beverage**	**α-Tocopherol (mg)**	**β-Tocopherol (mg)**	**Biotin (µg)**	**Calcium Pantothenate (mg)**	**δ-Tocopherol (mg)**	**Folic Acid Total (µg)**	**γ-Tocopherol (mg)**	**β-Carotene (µg)**	**Sum Tocopherols (mg** **)**	**Vit A (µg)**
15. Kenkey	0.092	0	2.92	0.049	0	11.7	0	0	0.092	NA
16. Shito sauce	13.8	0.577	5.2	0.172	2.43	9.22	10.8	78.7	27.7	0
17. Cassava and plantain fufu	0	0	0	0.199	0	6.63	0	0	0	NA
18. Malt/Malta drink	0.358	0	2.97	0.755	0	8.45	0	0	0.358	NA
19. Plantain chips	3.01	0	9.87	0.29	0	36.4	0	84	3.01	0
20. Eba (Gari)	0	0	0.124	0	7.68	0	0	0	0	NA
21. Rice and peas/beans	0	0	2.16	0.26	0.69	31	0.703	5.71	1.39	NA
22. Jollof rice	0.843	0	1.94	0.212	0	34.9	0.658	76	1.5	0
23. Egushi stew	1.55	0	5.51	0.273	0	33.6	2.37	897	3.92	0
24. Groundnut soup	1.89	0	16.1	0.214	0	35.4	1.18	359	3.07	0
25. Meat stew	2.51	0	6.22	0.128	0	15.9	2.23	132	4.74	0
26. One pot pepper soup	0.3	0	2.24	0.0887	0	8.67	0	41.2	0.3	0
27. Okro soup/stew	1.39	0	4.46	0.181	0	25.4	0	705	1.39	0
28. Ewedu soup	1.76	0	6.56	0.272	0	43.2	0	717	1.76	0
**Sample Dish/Snack/Beverage**	**Vit B1 (mg)**	**Vit B12 (µg)**	**Vit B2 (Riboflavin) (mg)**	**Vit B5 (Panthotenic Acid) (mg)**	**Vit B6 (Pyridoxine) (mg)**	**Vit C (mg)**	**Vit D2 (µg)**	**Vit PP/B3, (mg)**		
15. Kenkey	0.097	0	0.0466	0.0451	0.138	0	NA	0.622		
16. Shito sauce	0.032	0	0.131	0.159	0.121	NA	0	1.74		
17. Cassava and plantain fufu	0	0	0	0.183	0.0664	0	NA	0.319		
18. Malt/malta drink	0.297	0	0.0593	0.695	0.231	0	NA	1.58		
19. Plantain chips	0.024	0	0.0399	0.267	0.144	NA	NA	0.724		
20. Eba (Gari)	0	0	0	0.114	0	0	NA	0.191		
21. Rice and peas/ beans	0.055	0	0	0.239	0	NA	NA	0.483		
22. Jollof rice	0.043	0	0	0.195	0.0421	0	0	0.747		
23. Egushi stew	0.02	0.97	0.0283	0.251	0.0463	0	0	1.3		
24. Groundnut soup	0.039	0.938	0	0.197	0.0715	0.518	0	2.9		
25. Meat stew	0.034	0.803	0	0.118	0.0847	2.82	0	1.79		
26. One pot pepper soup	0.028	1.64	0.0221	0.0816	0.0563	0	0	1.52		
27. Okro soup/stew	0.036	0.962	0.0268	0.166	0.0595	0	0	1.33		
28. Ewedu soup	0.024	1.25	0.0624	0.251	0.107	2.35	0	1.6		

NA—Not Analysed.

**Table 13:** Vitamins composition of North African dishes, snacks and beverages in the UK (per 100 g edible portion).

**Sample Dish/Snack/Beverage**	**α-Tocopherol (mg)**	**β-Tocopherol (mg)**	**Biotin (µg)**	**Calcium Pantothenate (mg)**	**δ-Tocopherol (mg)**	**Folic Acid Total (µg)**	**γ-Tocopherol (mg)**	**β-Carotene (µg)**	**Sum Tocopherols (mg)**	**Vit A (µg** **)**
29. Chicken couscous	1.05	0	2.37	0.36	0	18.8	1.31	1446	2.37	0
30. Vegetable couscous	0.708	0	3.31	0.191	0	24.3	1.75	119	2.46	NA
31. Lamb couscous	0.54	0	3.18	0.248	0	18	1.41	660	1.95	0
32. Traditional Libyan soup	0.558	0	3.69	0.135	0	12.1	0.746	98.8	1.3	0
33. Kunafa	1.13	0	1.48	0.119	0	7.13	3.18	0	4.31	200
**Sample Dish/Snack/Beverage**	**Vit B1 mg**	**Vit B12 µg**	**Vit B2 (Riboflavin) (mg)**	**Vit B5 (Panthotenic Acid) (mg)**	**Vit B6 (Pyridoxine) (mg)**	**Vit C (mg)**	**Vit D2 (µg)**	**Vit PP/B3, (mg)**		
29. Chicken couscous	0.046	0	0	0.331	0.0714	5.72	0	2.01		
30. Vegetable couscous	0.063	0.492	0	0.176	0.0544	0.568	NT	0.798		
31. Lamb couscous	0.084	0.569	0	0.228	0.0779	1.57	0	0.782		
32. Traditional Libyan soup	0.029	1.09	0.016	0.124	0.0467	1.83	0	1.51		
33. Kunafa	0.032	0	0	0.11	0	0	0	0.387		

NA—Not Analysed.

## Supplementary Materials

The following are available online at https://www.mdpi.com/2304-8158/8/10/500/s1, Figure S1: Stages in the prioritisation of popular dishes, snacks and beverages., Figure S2: Composite samples preparation protocol, Figure S3: Stages in the preparation of composite samples.
