# Supplementary material for: Nutrient Composition of Popularly Consumed African and Caribbean Foods in The UK
Source: Foods. 2019 Oct 15;8(10):500. doi: 10.3390/foods8100500 (PMC6835955; doi:10.3390/foods8100500)
Supplement: Supplementary file 1 [file foods-08-00500-s001.pdf]

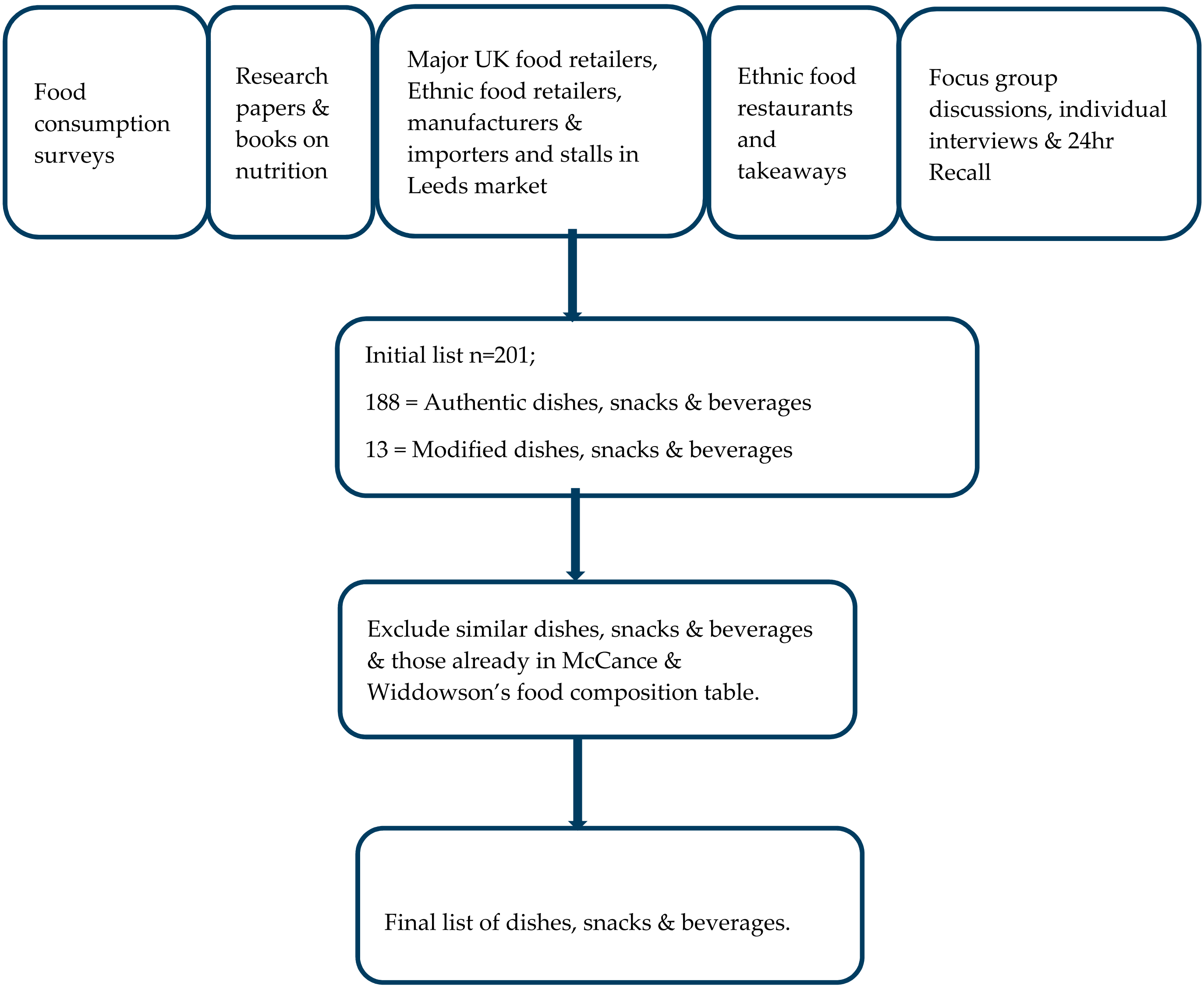

Figure 1 Stages in the prioritisation of popular dishes, snacks and beverages.

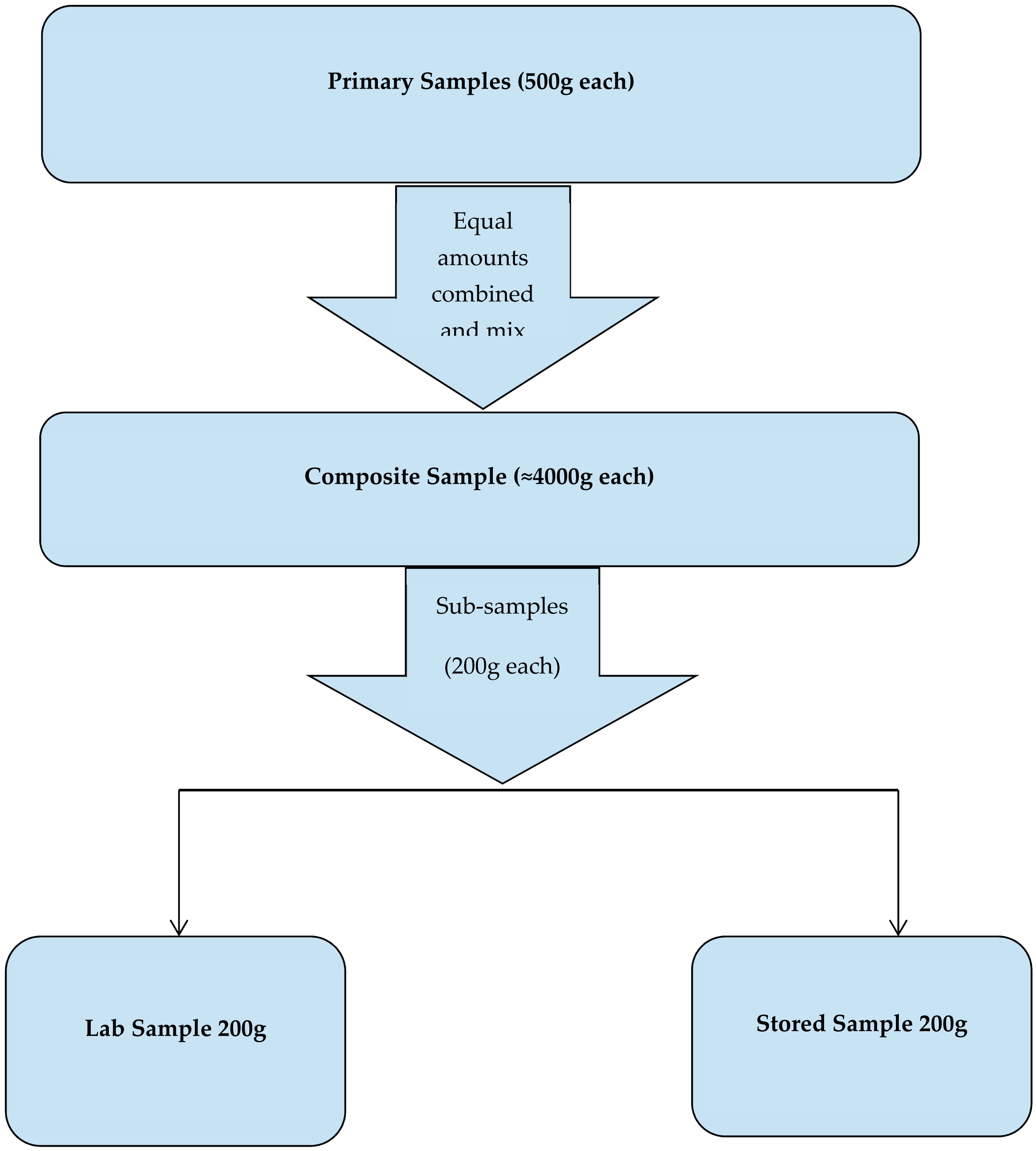

Figure 2 Composite samples preparation protocol

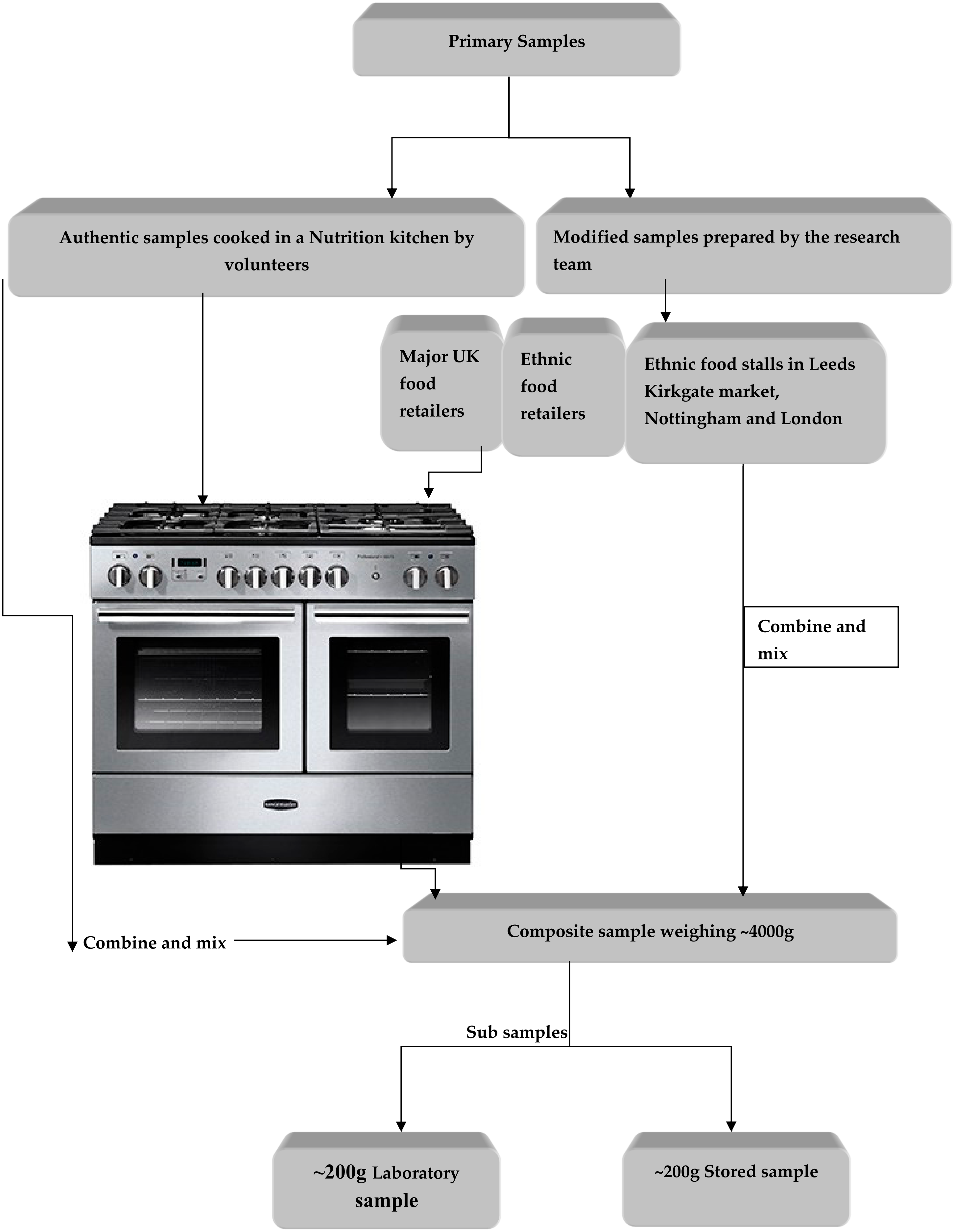

Figure 3 Stages in the preparation of composite samples
